# Fiber-integrated hydrogels: a versatile platform to improve structural and biological performance in 3D biofabrication

**DOI:** 10.1016/j.mtbio.2026.102799

**Published:** 2026-01-19

**Authors:** Annabelle Neuhäusler, Nils Lindner, Andreas Blaeser

**Affiliations:** a Technical University of Darmstadt, Institute for Printing Science, Darmstadt, Germany; b Technical University of Darmstadt, Center of Synthetic Biology, Darmstadt, Germany

**Keywords:** Hydrogels, Fibers, Tissue engineering, Bone, Muscle, Nerve, 4D-metamaterials

## Abstract

Hydrogels emerged as versatile biomaterials for tissue engineering due to their extra cellular matrix similarity and mechanical and biochemical properties. Still, hydrogels expose limited stiffness, anisotropy and nutrient diffusion. By reinforcing hydrogels with synthetic and natural fibers, these drawbacks can be effectively addressed, thereby enabling the modeling of advanced biomimetic tissue. This review discusses recent progress in the fabrication of fiber-integrated hydrogels and brings together developments from biomaterials, biofabrication, mechanobiology, and organ-model engineering. Fiber-addition impact on viscoelastic, time-dependent und nonlinear material properties, on multiscale and hierarchical constructs and on mechanical and biological readouts are analyzed. Specifically, the integration of both synthetic and natural fibers into hydrogel matrices is highlighted which significantly broaden their structural and biochemical versatility. These fiber-added hydrogels display improved properties including enhanced stiffness (up to 10-fold increase), anisotropy (>80 % alignment) and nutrient diffusion (4-fold increase). Moreover, the incorporation of fibers directly impacts cellular behavior by promoting adhesion, migration, proliferation and differentiation. Finally, bone, muscle and nerve tissue are exemplary presented in more detail to highlight the broad potential of these composite materials. In conclusion, fiber-embedded hydrogels represent a decisive step toward enhanced 4D-metamaterials.

## Graphical abstract

**Figure:**
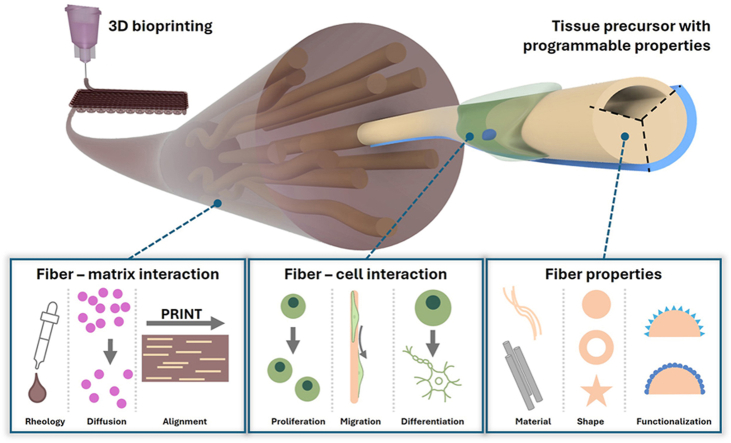

## 1 Introduction

Hydrogels are polymers with the ability to form porous, aqueous gels. They are outstanding in their use as a matrix material for tissue engineering due to their high resemblance with the native extracellular matrix (ECM) [1,2]. Despite their ideal growth conditions for cell culture, conventional hydrogels expose certain limitations such as the inherent mechanical properties of natural hydrogels which are often not sufficient to match the native stiffness [3,4]. One way to achieve mechanical reinforcement is to use elastic scaffold structures such as textile carriers or spacer fabrics [5,6] enabling the integration of continuous fibrous meshes. However, this limits the geometric freedom inherent in the processing of hydrogels using 3D-bioprinting. Another challenge in the use of bulk hydrogels is the lack of mimicking the anisotropy and directionality of native tissue [7]. Different approaches arose to overcome these drawbacks: dual crosslinking, supramolecular hydrogels or thermoplastic scaffolding [8]. The high degree of flexibility resulting from the manufacture and modification of the fibers [9], their independent integration, and the resulting possibility of adjusting the mechanical, anisotropic and diffusive properties have led to the increasing popularity of this modification method. For instance, poly lactic acid (PLA) short sub-micron fiber integration into alginate hydrogels yielded a threefold increase in Young's modulus which is similar to native cartilage [8,10]. It was moreover shown that the integration of short, single poly-caprolactone (PCL) fiber fragments into agarose hydrogels increased the passive diffusion limit of 300–500 μm up to 4-fold to 2000 μm for small biomolecules of 4 kDa [11]. Lastly, a vast number of publications is available showing the advantages of anisotropic fiber integration overcoming the limited anisotropy of bulk hydrogels. Exemplary, collagen fiber orientation regulates vasculature formation [12,13] and the alignment of electrospun fibers directly influences the self-renewal and differentiation potential of human mesenchymal stem cells [14]. 3D-bioprinting is an additive manufacturing method which allows the precise spatial deposition of hydrogel-based bioinks [15]. This leads to the production of structures with high resemblance to native tissue architecture [16] and holds great potential for automatization and personalization [17]. While extrusion bioprinting is a facile method to build anisotropic tissue properties by fiber alignment through the applied shear stress in the nozzle [18] it is currently limited regarding the integration of long, continuous fibers [19,20]. Ultimately, fiber-embedded hydrogels pave the way toward enhanced 4D-metamaterials which allow spatial-temporal transformation e.g. self-folding origami structures and cell-instructive behavior such as directional cell growth [21,22].

In clarification of the focus of this review, it is important to differentiate between **intrinsically fibrous hydrogels** and **fiber-loaded hydrogels**. Most protein-based hydrogels such as collagen or fibronectin expose a fibrillar structure which is usually in the range of nanometers [23]. Hereby, the native tissue architecture often cannot be matched, and the promotion of mechanical, diffusional or directional properties is limited. Conversely, this review focuses on the isolated production of fibers and their subsequent or simultaneous mixing with the hydrogel matrix supporting the active integration of fibers in 3D biofabrication workflows. This facilitates **the combination of two distinct material classes and unifies their benefits**, i.e. ideal growth environment of hydrogels with the functionalization through fibers. The multifaceted properties and requirements of hydrogels, fiber types, and biofabrication methods mentioned in the introduction cause an intricate interplay that can significantly influence the final tissue properties. To unravel this complex interaction, the article provides a comprehensive overview of common fiber types, their integration and effects on gel properties, and their influence on cell cultivation. Rather than proposing a theoretical design framework, the article summarizes the manufacturing possibilities of fiber integration and its influence on tissue engineering from a biofabrication perspective. Specifically, bioprinting is highlighted as a common biofabrication method and extensive discussion about other biofabrication methods such as casting, self-assembly or textile scaffolds can be found elsewhere [5,24,25].

## 2 Types of fibers used in hydrogels

Fiber types are commonly divided into synthetic and natural fibers based on their material origin (Table 1, Table 2) and thus applicable spinning methods and processing windows. Synthetic fibers are made of synthetic polymers such as poly lactic acid (PLA), polyvinylalcohol (PVA), polyethylenglycol (PEG) or polycaprolactone (PCL) (Table 1). Although they are inert in terms of cytocompatibility and biofunctionality, their mechanical properties are generally superior to natural polymers. Mostly, these polymers are handled using electrospinning or melt electro writing (MEW) [26]. The former is a widely used method where a polymer solution is introduced into an electrical field, thereby creating long, thin fibers which are collected on a flat or rotating spool collector resulting in a sheet-like fiber-mat [27]. Similarly, MEW utilizes an electrical field with the precise placement of fibers into 3D geometries although with a height limit [28]. A detailed description of other classical spinning techniques known from textile fabrication can be found elsewhere [5]. Fiber size of synthetic materials ranges in the micro-to nano scale. Notably, those fiber mats need to be fragmented to be pipettable and therefore used in bioprinting.

**Table 1:** Synthetic fiber materials.

Fiber material	Fabrication	Fiber diameter	Fiber length	Fiber %	Hydrogel	Cell type	Processing method	Mechanical data	Effect	Ref
Gold nanowires (GNWs)	n.a.	30 nm	4.5 mm	0.05 %	5 % Collagen	C2C12	Bioprinting	Youngs modulus -F 2.8 MPa + F(random) 4.5 MPa,+F(aligned) 5.4 MPa	Alignment (Guided growth)	[29]
PCL	Electrospinning	2 μm	<400 μm	0.10 %	0.5, 1, 1 % Agarose	HepG2, HUVECs	Bioprinting	Compression force + F 2-fold increase	Cell adhesion, viability, proliferation	[11]
	Melt electrowriting	9.7 ± 0.2 μm	∞	n.a.	0.75 % Hyaluronan-SH, 0.4 % Alginate, Gelatin, 0.45 % Matrigel	Neurons, astrocytes	Casting	No difference	Increased neuronal survival, neurite length, and neuronal firing	[28]
	Melt electrowriting	3.2 μm	∞	95 %	10 % GelMA	hMSC	Casting	Stiffness -F 70 kPa, +F up to 200 kPa	Increased mechanical properties, osteogenic differentiation	[30]
	Electrohydrodynamic printing	∼20 μm	not specified	not specified	3.5 % Gelatin, 2 % Fibrinogen	C2C12	Consecutive hybrid bioprinting	Modulus + F(aligned) 3 MPa, +F(random) 250 kPa	Generation of aligned myotubes	[20]
PCL (collagen-coated)	Electrospinning	not specified	not specified	2, 3, 5 %	2–3 % Spider silk protein eADF4(C16)	U87 cells, TdTomato-farnesyl expressing U87 reporter cells	Bioprinting	not tested	Printing dependent cell viability; increased cell elongartion	[31]
	Electrospinning	400–1000 nm	∞	n.a.	n.a.	hMSC	Seeding	not tested	Differentiation and alignment	[14]
PCL, collagen, graphene oxide	Electrospinning, Melt electrowriting	nm-μm	∞	n.a.	n.a.	Schwann cells, PC12	Seeding	multiscale structure 20-fold higher maximum stress	Electrical conductivity, cellular elongation and differentiation2	[32]
PCL, Gelatin-IGF1	Electrospinning, 3D printing	mm, nm	∞	n.a.	n.a.	MG-63, rMSC	Seeding	Emodul 17–35 MPa, compressive strength 2–3.4 MPa	Differentiation	[33]
PLA	XanoShear	<1 μm	<150 μm	1, 2 %	2.5, 3.5 % Alginate	Human chondrocytes	Bioprinting	Youngs modulus -F 7 kPa, +1 %F 11 kPa, +2 %F 25 kPa	High viability	[10]
PLA (Hydroxyapatite-coated)	not specified	∼40 μm	>500 μm	0.8–3.5 %	8 % GelMA, 2 % Alginate	MC3T3, HDF	Bioprinting	Storage modulus -F 1.3 kPa, +F 3 kPa	Cell adhesion, osteogenesis	[34]
PU	Electrospinning, aligned	nm	∞	n.a.	n.a.	C2C12, human iPCs	Casting	Emodulus fiber mats 10–50 kPa, Tensile strength 5–20 MPa	Adhesion, oriented growth, differentiation	[35]
PVA	Wet-spinning	nm-mm	∞	n.a.	PVA	n.a.	n.a.	Tensile stress isotrop 2 MPa, anisotrop 8 MPa	Anisotropic fiber development	[36]
PVA + carrageenan, PCL + propolis, PVA + fucoidan	Electrospinning, 3D printing	mm, nm	∞	n.a.	n.a.	L929, HUVEC	Seeding	Compressive modulus 270-240 kPa	Differentiation	[37]

n.a. = not applicable, -F = without fibers, +F = with fibers, +1%F = with 1 % fibers, +2%F = with 2 % fibers, PCL = poly-caprolactone, PLA = poly-lactid acid, PU = polyurethan, PVA = poly-vinyl alcohol, GelMA = Gelatin methacrylate, ∞ = continuous fiber.

**Table 2:** Natural fiber materials.

Fiber material	Fabrication	Fiber diameter	Fiber length	Fiber %	Hydrogel	Cell type	Processing method	Mechanical data	Effect	Ref
Alginate (cell-laden)	Co-axial spinning	50–200 μm	<5 mm	10–50 %	5, 7.5 % GelMA ("porogel")	HUVEC, human lung fibroblasts	Bioprinting	Compressive modulus -F ∼6 kPa, +F ∼5 kPa	Growth and spread of endothelial cells	[38]
Alginate and oxidized hyaluronan, cellulose, matrigel	Coaxial extrusion	Core: ∼300 μm, shell: ∼600 μm	∞	n.a.	n.a.	Neuronal stem cells	Cell encapsulation	not tested	Anti-inflammatory effect, guided axon regrowth	[39]
Alginate, GelMA	Coaxial microfluidic spinning	∼500 μm	∞	n.a.	n.a.	HUVEC, PC12	Cell encapsulation, 3D printing	not tested	Diffferentiation, barrier effect of HUVECs	[40]
Alginate, cellulose	Multifilament 3D bioprinting/chaotic printing	1 mm	∞	37–50 %	3 % GelMA, 3.5 % Alginate	C2C12	Bioprinting	E-modul + F 4–25 kPa	Cell proliferation, spreading and alignment	[41]
Cellulose	Self-assembly	nm	∞	0.005, 0.01, 0.5, 1 %	1, 2, 3, 4 % Alginate, 1, 2, 3, 4 % Carboxymethylcellulose	Porc1	Bioprinting	not tested	Shear thinning, higher viscosity, high cell viability	[42]
	Hydrolysis	nm	∞	1, 2, 3 %	3.3 % Alginate, 6.7 % Gelatin	ATDC5	Casting	E-modul -F 13 kPa, +1 %F 17 kPa, +2 %F 20kPA, +3 %F 9 kPa	Differentiation	[43]
	Oxidization	nm	∞	10–40 %	2 % Alginate	Fibroblasts	Casting	Compressive modulus -F 90 kPa, +10 %F 100 kPa, +20 %F 160 kPa,>20 %F decreasing	Higher viability and proliferation	[44]
	Extraction	nm	∞	0.80 %	*N*,*N*-dimethylacrylamide or *N*-isopropylacrylamide	n.a.	Bioprinting	Youngs modulus longitudinal 1200 kPa, transverse 1000 kPa	n.a.	[21]
Cellulose, PCL	Extraction (Cellulose), Electrospinning (PCL)	Cellulose: 14 nm, PCL: 3 μm	Cellulose: ∞, PCL: aspect ratio 15	Cellulose: 0.5,1,2 %; PCL: 2,5,10 %	3 % Alginate, 25 % Pluronic	n.a.	Bioprinting	not tested	Increased viscosity, increased shape fidelity	[45]
Collagen	Electrospinning	Ratio 4-46	Ratio 4-46	0.50 %	3 % Hyaluronan	C28/I2	Injection	Compressive modulus -F 6kPA, +F 8kPA	Cell survival	[46]
	Microfluidic spinning	4 μm	<500 μm	25, 50 %	n.a.	HepG2	Seeding	not tested	Spheroid formation, increased albumin	[47]
	Aspiration-ejection method	μm	∞	5–12 %	0.2 %Collagen	hBMMSCs	Casting	Ultimate tensile strength increase with increase fiber ratio 40–300 kPa	Tenogenic, chondrogenic or osteogenic differentiation, high cellular alignment	[48]
	Homogenization or sonication	<27 μm	<200 μm	2 %	1 % Fibrinogen, gellangum	HDF, HUVEC	Bioprinting	not tested	Lumen formation, alignment along collagen fibers	[49]
	Microfluidic spinning	5–50 μm	<200 μm	0.50 %	0.5 % Agarose, 0.5 % Hyaluronan	C2C12, PC12, hMSC	Bioprinting	Emodul -F 20 kPa, +F 12 kPa	Network formation, differentiation, cellular guidance	[22]
	Wet-spinning	50–200 μm	∞	n.a.	n.a.	Dorsal root ganglion (DRG) neurons and Schwann cells	Seeding	Tensile moduli lowest collagen (0.75 %) 1200 MPa, decreasing to 130 MPa in 3.4 % collagen	High cell viability, high neurite outgrowth	[50]
Collagen (cell-laden)	Microfluidic spinning	250 μm	∞	n.a.	n.a.	PC12, rat aortic endothelial cells	Culture	not tested	Guided growth	[7]
Gelatin	Solution blow spinning	25–50 μm	250 μm	100 %	n.a.	MC3T3-E1	Seeding	not tested	Increased osteogenic activity	[51]
	Rotary jet spinning	4.20 ± 0.23 μm	<1 mm	5–10 %	2.4 % Gelatin, 2.4 % Alginate	Neonatal rat ventricular cardiomyocytes (NRVM)	Bioprinting	not tested	Cellular alignment along fiber, cyclic contractility	[52]
Gelatin, albumin and hemoglobin	Microfluidic spinning	10–60 μm	1 mm	25 %	0.7 % Alginate	HepG2, Swiss-3T3 (fibroblasts)	Casting	Youngs modulus 50 kPa	Cell adhesion, viability, proliferation	[47]
GelMA, Alginate	Coaxial bioprinting	200–600 μm	mm	n.a.	GelMA	HUVECs, tumor	Bioprinting	E-modulus -F 4 kPa, +F 6 kPa	Higher viability and alignment	[53]
NorHyaluronan, NorGelatin, GelMA, Hyaluronan-MA	Electrospinning	<1 μm	<20 μm	20, 50, 75 %	3.5 % NorHyaluronan, 0.1, 0.25, 0.6 % collagen-I	hMSC	Bioprinting	G' increase with lower fiber concentration	Contractile microtissues	[54]
Peptide-nanofibers	Self-assembly	nm	μm	4 %	4 % Polypeptides	HUVECs, neuronal cells	Casting	not tested	High cytocompatibility, self-healing	[55]
Silk microfibers	Hydrolysis	3–5.5 μm	50–150 μm	1, 3, 5 %	5, 6, 7 % Silk fibroin, 2 % Gelatin	C2C12	Handheld bioprinting	Youngs modulus -F ∼8 kPa, +1 %F ∼15kPA, +3 % and +5 %F ∼20 kPa	Cellular alignment	[56]
	Grinding	100–300 nm	4–7 μm	1 %	1.5 % Hyaluronan	Mammalian cells	Bioprinting	Youngs modulus -F 1 kPa, +1 %F 2.3 kPa	Cell viability >85 %	[4]
	Extraction	nm	∞	1, 2, 3 %	5 % Alginate	L929	Bioprinting	Tensile strength -F 70 kPa, +1–2 %F up to 150 kPa, +3 %F 25 kPa	Cell survival, proliferation and spreading	[57]
Silk, polyester, steel	Long fiber embedding (FRESH)	μm	μm	not specified	4 % Alginate, 0.6 % Collagen	n.a.	FRESH	Tensile modulus -F 50 kPa, +F 380 kPa, tensile strength -F 10 kPa, +F 45 kPa	Structural reinforcement	[19]

n.a. = not applicable, -F = without fibers, +F = with fibers, +1%F = with 1 % fibers, PCL = poly-caprolactone, GelMA = Gelatin methacrylate, ∞ = continuous fiber.

On the other hand, natural fibers are made from naturally derived polymers such as collagen, gelatin, silk or nanocellulose [58] (Table 2). Natural polymer fibers are superior in terms of cell-compatibility and biofunctionality and are therefore of great interest in producing highly cell-instructive matrices. However, a high batch-to-batch variability is inevitable, and their handling and processing requires caution due to their sensibility towards temperature, degradation and shear [59]. For instance, collagen as a native ECM protein [60] as well as algae-derived polysaccharides such as alginate are mostly spun using wet-, microfluidic- or even electrospinning [61]. Wet and microfluidic spinning take advantage of laminar flow dynamics and pH-, ionic or UV-induced crosslinking to yield a continuous fiber [62]. Fiber diameters range from several to hundreds of micrometers. Similarly to synthetic fibers, a subsequent fragmentation step is needed for bioink integration of hydrogel fibers. Other natural polymers like silk or nanocellulose are harvested from silkworm cocoons, bacterial culture or plant-based substrates. Their size range is in the nanometer scale, and they are easily integrated into hydrogels without further fragmentation [4].

Apart from single-origin fibers, a variety of composite fibers exist, either made up of multiple polymers or (bio-)polymer coatings. The former requires more complex spinning set-ups, for example coaxial needle designs for electrospinning of core-shell fibers [11]. Here, additional substances as growth factors or drugs can be incorporated and thus apply for drug or protein delivery assays [63,64]. Microfluidic spinning is another technique used for multiple component fibers where a multitude of channels and crosslinking methods need to be united. For instance, a multi-material, 16-layer fiber composed of alginate, GelMA and cellulose was created by implementing so-called chaotic spinning which is an adapted microfluidic spinning technique [41]. As such an approach is laborious and error-prone [65,66], coatings can be an easier way to provide cell attractive cues as shown by a study where cellular adhesion was improved by coating PCL fibers with collagen [14].

Lastly, different fiber sizes and compositions can be combined to generate multi-material, multi-scale and hierarchical fiber scaffolds. In multi-scale models, several size ranges are combined to harness a broad range of functionalities and reinforcement. Nanoscale fibers provide cues on a cellular level (e.g. adhesion), while micro and macroscale fibers enhance mechanical properties of the composite structure. For instance, the group of Al-Hammoud designed a three-level scaffold of multiple size ranges: the middle layer was composed of a 3D printed PVA scaffold with fiber diameters of 500 μm. The bottom and top PCL layers were each electrospun onto that scaffold with fiber diameters in the nanometer range [37].

To sum up, fibers produced from synthetic materials benefit mechanical enhancement of hydrogels while showing limited cell-instructive and biofunctional properties. On the other hand, natural polymer fibers show extraordinary biofunctionality. Their production is often more sensible to external factors, and their mechanical properties match soft tissues rather than reinforcing structures. Notably, a growing body of research combines multiple fiber sizes to create multi-scale and hierarchical tissue mimicry.

## 3 Strategies for fiber integration in bioprinted hydrogels

Due to the variety of biofabrication techniques, there is a need for various strategies for fiber integration into hydrogels. Specifically, classical approaches such as 3D printed polymer scaffolds or electrospun fiber mats are commonly seeded with cells or a hydrogel is casted on the structure. Conversely, bioprinting demands a pipettable bioink where fiber fragments are evenly dispersed in a hydrogel. Consequently, fibers need to be in a fragment size range so that a pipette or extrusion needle remains unclogged.

**Fig. 1:**
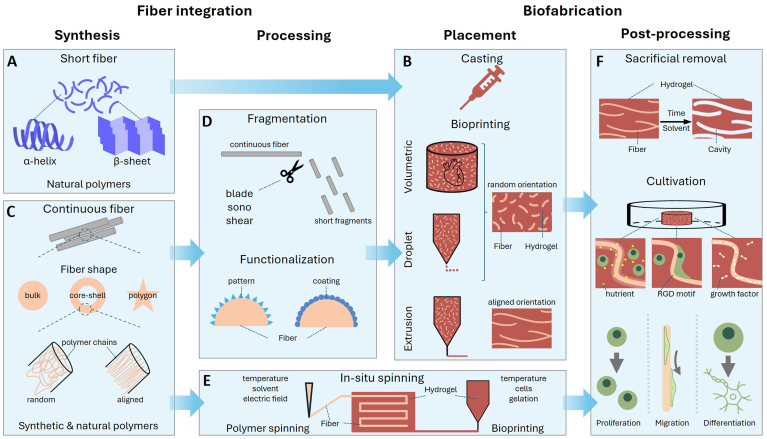
Methods for fiber integration. A As-produced short fibers in the nanoscale have alpha-helical or beta-sheet conformation. B Fiber-integrated bioinks can be either casted or bioprinted with volumetric, droplet-based or extrusion bioprinting. Extrusion allows aligned fiber orientation. C Continuous fibers in the microscale can have different shapes, ranging from round bulk and core-shell to polygonal shapes. By stretching, polymer chains within fibers are aligned. D An extra processing step is needed to generate a pipettable bioink: fragmentation by blade, sono or shear. Additionally, functional coating or plasma treatment can be applied for cell attractant effects. E Fibers in the micro-to macroscale can be implemented by an in-situ spinning approach where hydrogel and fibers are spun and printed simultaneously. F Post-processing steps as sacrificial fiber removal and cultivation. Fiber integration increases cellular proliferation, migration and differentiation.

There are two distinct synthesis approaches to fiber fragmentation: either fibers are already in a short, single fragment condition or are produced as a continuous thread (Fig. 1). Nanofibrous materials such as cellulose, silk or polypeptides are examples of the former (Fig. 1A). They are in general easier to produce or can even be purchased as such. Their nanofibrillar structure is formed by self-assembly and no extra processing step is needed to produce short fiber fragments. Such bioinks are suitable for casting and bioprinting techniques followed by a cultivation step for tissue maturation (Fig. 1E and F). For instance, silk as a natural polymer gained attention especially in medicine due to its high cytocompatibility, easy processing and abundant supply from the mature sericulture industry [4]. It is produced by silkworms and subsequent degumming, grinding and purification. Their main advantages were found to be a mechanical increase of the bioink, higher shape fidelity and the support of muscle tissue engineering [4,56]. Likewise, nanocellulose is often produced by bacterial culture, forms nanofibrils by self-assembly and exposes diameters in the nanoscale [67]. Its percentage was tested in a wide range: from a few percentages inducing higher viability and proliferation up to 17 % for cartilage regeneration *in vivo* [67,44]. Interestingly, protein polymers with a beta-sheet or alpha helical conformation can be designed and synthesized to target cell adhesion and differentiation [55].

Nevertheless, most spinning techniques produce continuous fibers in the micro-to millimeter scale and need to undergo several processing steps such as spinning, fragmentation, functionalization and resuspension to yield a pipettable bioink (Fig. 1B). Continuous fibers are frequently produced by electrospinning, MEW, rotary jet spinning and wet- or microfluidic spinning [10,11,52,68]. The latter two methods even allow for the incorporation of living cells [7]. Importantly, to incorporate continuous fibers or fiber mats into a bioink, a fragmentation step is obligatory such as cryogenic [69], ultrasonic [70], motor-driven blade cutting [71], UV cutting [72] or shear-induced fragmentation [54] (Fig. 1C). Such bioinks are appropriate for casting and bioprinting, although they require a more laborious production process. As an additional processing step, fiber coatings and functionalization can be used to tailor cellular response as well as fiber-matrix bonding. For example, plasma treatment modifies a polymer's surface by introducing hydroxyl, carboxy or amine groups to the fiber surface which promotes wettability and adhesion [73]. Limited cell adhesion and proliferation due to hydrophobic surface characteristics can furthermore be overcome by utilizing functional coatings. For example, collagen adsorbs onto fiber matrices which provides bioactive sites for cell attachment [74].

An interesting exception to the previous two cases is so-called in-situ spinning (Fig. 1D). This process enables the controlled integration of continuous, aligned fibers into three-dimensional hydrogel constructs. Although the fiber volume fraction usually remains small, this technique is particularly interesting as microscopic fibers can be integrated into 3D structures without laborious processing steps. This technique is highly demanding in terms of hardware design and implementation as the applicable process temperatures are often incompatible, e.g. PLA 220 °C and collagen 10 °C [75,34]. Sun et al. reported such an intricate set-up with which they created alginate-collagen structures supported by long embedded silk or polyester fibers. The resulting construct showed a tensile modulus of about 400 kPa, which is a 4-fold increase to the bulk hydrogel [19].

Lastly, the solving of the fiber material post-processing after printing or casting is an interesting technique similar to sacrificial bioprinting (Fig. 1F). Hereby, empty cavities are created by first mixing fibers into the hydrogel matrix followed by gelation and subsequent dissolving. These cavities can act as a type of vasculature for nutrient transport or can even directly be seeded with cells. To illustrate, Guo et al. created cell-laden alginate microfibers, integrated those into a GelMA hydrogel and subsequently resolved the fiber material [38]. Thereby, hollow, cell-laden cavities were created, enabling structured vascularization. Going even a step further, Cavero-Arrivasplata and colleagues adapted a microfluidic spinning set-up to spin multi-material fibers which incorporate a reinforcing alginate layer, a myoblast-laden GelMA layer as well as a sacrificial layer for nutrient supply within the fiber [41]. By removing the sacrificial cellulose layer, empty cavities inside the fiber itself were produced, supporting efficient nutrient supply and increased long-term viability.

In brief, directly implementing as-produced fragments is straightforward and compatible with all biofabrication methods, although fibers often expose undefined length or diameter and show high batch-to-batch variability similar to other natural materials. On the other hand, continuous threads have a defined spinning process with fewer fiber variety although an extra fragmentation step is needed. Such fibers can be functionalized by coatings or are inherently multi-material, and some methods even allow the incorporation of living cells within the fibers. However, the process is more laborious, and materials can be sensitive to their environment. Fragmented fibers are suitable for most 3D bioprinting biofabrication methods. Lastly, in-situ bioprinting is an interesting emerging technique enabling the embedding of long fibers which excel in mechanical properties as tensile and compressive strength. However, the intricate printer design and varying processing windows of different materials need to be taken into account.

## 4 Impact of fiber integration in bioprinted hydrogels

Incorporating fibers into hydrogels unlocks a spectrum of effects. The resulting composite material acts as a complex, tunable material. Depending on the fiber type, orientation and distribution, these composite materials exhibit distinct physical, mechanical and biological properties. The following discussion will illuminate how fibers influence printability and structural integrity, mechanical reinforcement as well as cellular response.

### 4.1 Printability and structural integrity

**Fig. 2:**
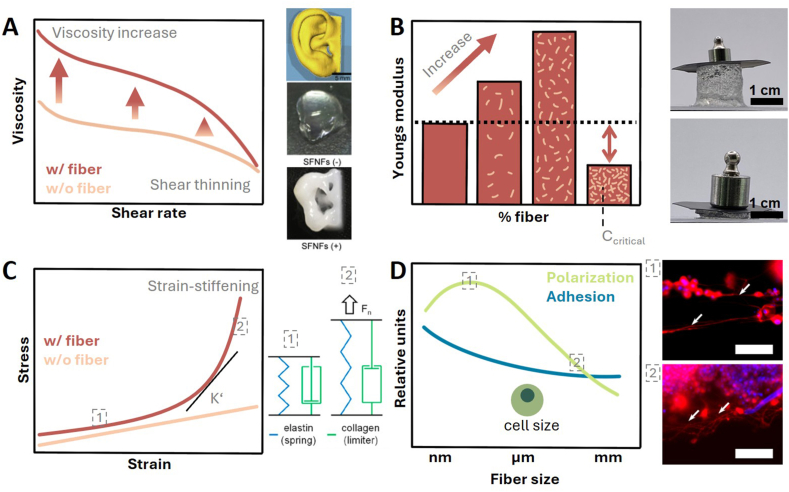
Impact of fiber integration on bioprinted structures. A Viscosity increases with fiber integration while maintaining shear thinning behavior (left). This is also reflected in a higher shape fidelity post-printing (right). SFNFs = silk fibroin nano fibers, scale bar = 1 cm. Adapted from Ref. [4]. B Fiber added hydrogels are reported to be mechanically reinforced, reflected by the increase in Young's modulus until a certain fiber volume fraction (C_criticlal_) when the construct's modulus decreases drastically. Adapted from Ref. [11]. C Strain-stiffening is a common motif in native tissue as arteries or bone. Fiber addition to bulk hydrogels could mimic this effect (left). K’ = differential modulus. Elastin functions as a spring (inlet 1) while collagen acts as a limiter (inlet 2) in native tissue (right), adapted from Ref. [81]. D Cellular polarization and adhesion are influenced by fiber size. Inlets 1 and 2: smaller fiber diameters promote increased alignment of neurites of PC12 cells while larger fiber diameters lead to unorganized neurite outgrowth. Scale bars 100 μm. Adapted from Ref. [22]. w/fiber = with fiber, w/o fiber = without fiber.

When using bioprinting as a biofabrication method, hydrogels are often utilized as the base matrix material. They expose viscoelastic behavior, i.e. they show solid (elastic) as well as liquid (viscous) material characteristics. Therefore, viscosity, shear modulus, thixotropy and yield stress are important parameters to investigate [76]. Behavior upon shear ranges from shear-thickening (more viscous upon shear) to Newtonian (same viscosity upon shear) and shear-thinning (more fluid upon shear). Shear-thinning behavior is desired during bioprinting as it minimizes cell damage during high shear stress periods [77] while the desired viscosity depends highly on the printing technique: extrusion bioprinting is only feasible with higher viscous fluids whereas droplet-based bioprinting enables printing of low-viscosity hydrogels [78,79] (Fig. 2A, top). It is generally accepted that the integration of non-colloidal particles such as fibers increases shear-thinning behavior [80], however, a higher fiber volume fraction may promote aggregation and clogging, predominantly disadvantageous in extrusion-based applications or casting. In line with that, Schaefer et al. reported an increase in shear-thinning behavior as well as viscosity by integration of 3 % PCL fiber fragments into spider silk bioink [31]. Likewise, Sonnleitner et al. showed that the integration of either 10 % PCL microfibers or 2 % cellulose nanofibers into alginate hydrogels increases the shear-thinning behavior as well as the viscosity 40-fold (PCL) and 80-fold (cellulose) [45]. Interestingly, this effect is also dependent on the hydrogel: the same fiber addition to pluronic led to a viscosity increase of only 2-fold (PCL) and 4-fold (cellulose).

Thixotropy is the time-dependent ability of a material to return to its initial viscosity after a period of high shear. Shape fidelity benefits from fast thixotropy time, since the initial (higher) viscosity is reached faster and is therefore suitable to hold a printed shape efficiently. Conflicting results exist in this area as fiber integration was reported to prolong the recovery time [22] as well as decrease recovery time [42]. One study invested this discrepancy by incorporating either nanocellulose or PCL fibers into alginate and pluronic hydrogels. While nanocellulose addition resulted in a better shape fidelity for both hydrogels, PCL fiber addition to the pluronic hydrogel led to a time-dependent decrease in shape fidelity. Nevertheless, fiber integration into bioinks was consistently reported to improve the shape fidelity of 3D printed constructs, whether using cellulose, silk, or synthetic materials. For instance, a higher shape fidelity is yielded by integrating silk fibers, represented by smaller line widths compared to non-fiber hydrogels [4,56] (Fig. 2A, bottom). A similar result was found for PCL and PLA fiber integration into agarose based bioinks [11,82]. Together, these findings show the ambiguous influence of fiber integration on composite properties and highlight the importance of fiber-matrix compatibility. Ensuring optimal integrity of the composite material is crucial for the overall performance of the material. Fiber-matrix compatibility is dependent on the characteristics of both materials and different mechanisms influence the bonding quality [83]. First, the fiber's properties such as surface chemistry, roughness or geometry influence the micromechanical interlocking of fiber and matrix as well as the failure modes at the interface such as debonding, pull-out and shear [84]. Second, at the molecular fiber-matrix interface, chemical bonding, physical entanglement, and frictional interactions are predominant. Fiber and matrix can either be non-covalently bonded by van-der-Waals and electrostatic interactions or coupled over covalent bonds with a crosslinking agent [[85], [86], [87]]. Surface functionalization can be used to improve fiber-matrix bonding. Physical functionalization like laser ablation or etching improves fiber-matrix bonding by altering the surface geometry [88,89]. Chemical functionalization results in an altered surface composition respectively introduction of new functional groups. Common examples are plasma treatment, self-assembled monolayers and electrochemical deposition [74,90]. In conclusion, fiber-matrix bonding is maximized by increasing wettability, interfacial bonding and interface area size.

Native tissues are fibrous and anisotropic, i.e. the fibrous network is directional to support cell signaling, guidance and force distribution. To achieve anisotropic properties by the alignment of integrated fibers, extrusion bioprinting is a suitable method. In fact, it was found that the alignment of integrated fiber fragments during shear is mostly dependent on the ratio of their long and short axis [91]. In line with that, many studies report the successful alignment of fibers during extrusion bioprinting [11,21,22,34]. As an additional effect, fiber alignment can entail the subsequent cell alignment and oriented tissue formation. Exemplary, one study found that integrated gelatin fiber fragments could be aligned using extrusion printing and subsequently lead to the formation of anisotropic cardiomyocyte tissue [52]. Besides, swelling properties can be influenced by the anisotropy of the material to fabricate programmable architectures which change their shape in a time-dependent manner [21].

Overall, fiber integrated bioinks are suitable for bioprinting due to their shear-thinning behavior and high viscosity leading to increased shape fidelity. Although caution needs to be taken in choosing a compatible fiber-matrix combination. Resulting from shear stress inside the nozzle, the alignment of fibers inside a printed structure is feasible and leads to subsequent anisotropic tissue formation.

### 4.2 Mechanical reinforcement

The weak mechanical properties of hydrogels can be actively strengthened by exploitation of the fiber's intrinsic properties, i.e. high strength and impact resistance [87]. Specifically, the fibers' capacity to partly absorb and dissipate mechanical forces can be valuable regarding matrix strength, stiffness and elasticity. Many authors reported the increase in the e-modulus, tensile modulus or strength with increasing fiber volume fraction (Fig. 2B, top). For instance, Kamaraj et al. report an almost threefold increase in the Youngs modulus of silk fibroin hydrogel with 3 % silk microfibers compared to the bulk hydrogel [56]. Likewise, Mohammadpour et al. report that adding 2 % silk nanofibrils to an alginate matrix increases the elastic modulus threefold, boosts tensile strength fivefold, and improves compressive strength 2.2-fold [57]. Another study found that cellulose-filled alginate hydrogels exhibit increased stiffness, with a maximum compressive effective modulus of ∼20 kPa compared to ∼13 kPa in the bulk hydrogel [43]. However, there seems to be a fiber volume fraction cutoff at which E− and tensile moduli decrease drastically, often lower than the bulk control [57,43] (Fig. 2B, C_critical_). For example, the integration of more than 20 % nanocellulose in alginate hydrogels leads to a drop in compressive modulus [44]. These findings suggest that the force dissipation of fibers within hydrogels cannot be exploited infinitely. Integrating an excessive amount of fibers leads to more fiber-fiber than fiber-matrix interactions, as well as microstructural defects. Hence, the compatibility between fiber and matrix is crucial for stress transfer and performance. The strength of the fiber-matrix interaction is mainly dependent on fiber wettability, interfacial bonding and the matching mechanical properties of fiber and hydrogel [87]. Long fibers benefit from almost pure mechanical dissipation on the fiber, while the gel bears nearly no load. Short microfibers realize their load bearing performance on a more effective hydrogel-fiber interaction. Nanofibers dissipate the load optimally due to the maximized fiber-hydrogel surface area and thus high fiber-hydrogel bonding.

An additional aspect of mechanical reinforcement is the alignment state of fibers within the hydrogel construct. This is already a known fact for fibrillar alignment within fibers: by stretching a wet-spun PVA fiber, the tensile stress increases 4-fold compared to an isotropic fiber [36,92]. A similar mechanism is reported for fiber integrated hydrogels. The group of Kim et al. aligned gold nanowires magnetically in a collagen hydrogel and found the Young's modulus increased from 4.5 MPa (unaligned) to 5.4 MPa (aligned) [29]. With an even stronger effect, highly aligned PCL fibers in a gelatin-fibrinogen hydrogel increased the modulus from 250 kPa (unaligned) up to 3 MPa (aligned) [20].

Lastly, multiple size ranges and fiber-matrix interactions can be combined to generate multi-scale fiber reinforcement. Fang et al. developed an electrospinning and MEW method to create a composite scaffold ranging from the nano-to micrometer scale. This composite scaffold showed a 20-fold higher maximum stress compared to each individual structure suggesting a synergistic effect of both materials and size ranges [32]. Another interesting approach was done by using a 3D printed PVA scaffold in the micrometer range. This scaffold was used as a collector for two electrospun PCL layers with fiber diameters in the nanometer range. This multi-scale structure had superior tensile modulus (20 MPa compared to 15 MPa only 3D printed) as well as a fast epithelialization *in vivo* [37]. Beyond that, integration multiple fiber size ranges within a single composite construct offers a promising strategy to mimic the hierarchical, strain-stiffening behavior of native tissue [93] (Fig. 2C). Conversely to the linear behavior of many biomaterials, strain-stiffening materials show a constant stiffness at small deformations, however when a certain force is exceeded, a strong nonlinear stiffening is seen [81]. Comparable to the E− or shear modules in linear regimes, the differential modulus K’ is calculated from the slope in a stress-strain curve in the nonlinear regime [94] (Fig. 2C). For instance, strain-stiffening is known from blood vessels, mesentery tissue, lung parenchyma, cornea, and cortical bone [95]. In vitro, the strain-stiffening magnitude is dependent on several parameters such as fiber density, aspect ratio or orientation [96]. Moreover, fiber-matrix compatibility plays a crucial role as a weak bond leads to early delamination and therefore no complex, nonlinear behavior can be seen [86]. Exemplary, one study warp-knitted two distinct materials to mimic collagen and elastin polymer behavior in native arteries. Non-elastic TPU represented elastin as the “spring” and elastic PVDF (polyvinylidene fluoride) represented collagen as the “limiter” (Fig. 2C, right). Thereby, a superior strain-stiffening material was developed which closely mimics the mechanical properties of the native ECM in arteries [81]. Another recent study investigated the strain-stiffening of dextran-filled pectin and underlined the importance of filler concentration and filler-polymer interaction strength [96]. Although the cited studies focused on either continuous fibrous networks or colloidal fibrillar assemblies, the underlying mechanics suggest that integrating fibers of different diameters and aspect ratios into a bulk hydrogel would produce comparable nonlinear reinforcement. This is particularly plausible when the interfacial coupling between fibers and matrix is sufficiently strong to enable efficient load transfer [97].

Taken together, fiber integration benefits mechanical reinforcement of hydrogels by absorbing and dissipating forces. This effect is maximized when fiber and hydrogel are compatible, although a fiber volume fraction cut-off exists where mechanical properties drop drastically. Apart from that, fiber alignment increases directional mechanical improvement. Multiple size ranges of fibers can be combined to create superior scaffold characteristics, generating hierarchical scaffolds and mimic strain-stiffening behavior of native tissue.

### 4.3 Cellular response

Integrating fibers into bioinks can replicate the fibrous and hierarchical architecture of the native ECM *in vitro* which is crucial for optimal tissue function. The fiber's structural, mechanical and biochemical cues present adhesion sites, guide cell migration, and influence differentiation. Cells can sense their environment by integrin binding to adhesion motifs such as the RGD sequence or topographical cues and subsequent actin cytoskeleton extension which is called mechanotransduction [98] (Fig. 2C, top). In combination with a hydrophilic and patterned surface chemistry cell adhesion excels [99].Exemplary, human mesenchymal stem cells were able to reorganize and contract on gelatin fiber mats which expose binding motifs, a hydrophilic and patterned surface [54]. Cellular behavior is not only influenced by fiber porosity, topography and surface chemistry. Fiber diameter, alignment, and mechanical properties play a crucial role as well [100]. Notably, these factors can influence each other, increasing the complexity of potential interactions. Nanoscale fiber diameters enhance cell adhesion, while microscale fiber diameters promote cell elongation. Interestingly, significantly larger or smaller diameters than cell size lead to reduced polarization and guided migration due to lacking curvotaxis [100] (Fig. 2C, top). Therefore, a restricted size range seems to exist for optimal cellular response. Saeki et al. were able to show such a size preference of hepatocytes: by combining bio-inert alginate hydrogels with gelatin microfibers, they created stable 3D constructs with increased cell proliferation when using 4 μm fibers compared to 20 μm [47]. Another study found that neurite formation of PC12 cells depends on collagen fiber size. Smaller fiber diameters of 5–10 μm promoted significantly longer neurite outgrowth whereas larger diameters of >50 μm enhanced randomly oriented, branched neurites [22] (Fig. 2C, bottom). Furthermore, fiber alignment strongly influences cellular polarization and migration behavior. Consequently, it was found that aligned fibers promote polarization along the fiber as well as overall higher migration rates which supports anisotropic tissue formation. Collagen as the main component of the native ECM was used to guide cellular growth in many studies. Exemplary, PC12 cells showed over 80 % oriented spreading along the main axis of aligned collagen microfibers compared to the collagen bulk hydrogel [7]. The alignment of collagen was moreover shown to be beneficial in the formation of 3D vascular networks and lumen [12]. This hints at an elevated cell migration of endothelial cells as well as enhanced mechanosignaling. In another study, the effect of alignment and porosity of PCL fiber meshes on stem cells was investigated [14]. The authors reported that adipogenic and osteogenic differentiation was promoted by small pores and a random organization compared to increased stemness with larger inter-fiber distance. This study points to another important factor when considering fiber characteristics, namely fiber-fiber distance. Apart from that, the differentiation of progenitor cells into mature tissue is moreover dependent on the stiffness of the material. In this vein, chondrogenic differentiation was achieved by elevating the stiffness of an alginate-gelatin hydrogel by integrating nanocellulose fibers [43]. Similarly, the osteogenic differentiation of MC3T3-E1 was enhanced by stiff gelatin microfiber [51].

Almost all studies on fiber-integrated hydrogels found an increase in viability [11,42,47,53]. For instance, Schaefer et al. showed an increased cell viability of casted PCL fiber-added bioinks compared to regular hydrogels [31]. Similarly, nanocellulose functionalized alginate yielded an increase in viability of encapsulated fibroblasts [44]. However, the underlying mechanism has not been illuminated. One study hypothesized an increased passive diffusion by hollow PCL fiber integration into agarose hydrogels due to their similarity to vasculature [11]. However, compared to a bulk fiber integrated control, nutrient diffusion in hollow fiber samples was not altered. The diffusion increase could therefore be explained by a small water film on fiber-matrix interfaces upon agarose gelation, creating a small space for nutrients to diffuse freely. This would fit other theories that the fibers increase the porosity of the hydrogel network and therefore ease nutrient diffusion into and out of the structure [101].

Together, fiber integration affects cellular behavior in a complex manner. Surface characteristics such as hydrophilicity, micro- and nanopattern and coatings influence cell adhesion. Fiber size, alignment and density influence cellular migration and orientation. Inherent mechanical properties can strongly influence cell fate in terms of proliferative or differentiative outcome. Lastly, fiber integration slightly disrupts the innate polymer network of the hydrogel and thus increases matrix porosity, leading to improved nutrient diffusion and overall higher cell viability.

## 5 Applications in tissue engineering

Fiber integrated hydrogels and scaffolds expose significant improvement for various *in vitro* tissue types: vasculature, skin, liver or stomal tissue have been reported [57,47,102,49]. However, this chapter aims to focus on the advantages of fiber integration in three distinct applications: Bone and cartilage tissue, cardiac and skeletal muscle tissue as well as neural tissue. Thus, the broad application of various fiber types is implied: from stiff scaffold engineering for bone and cartilage, to aligned fibers and guided cellular growth for muscle tissue toward electrically conductive fibers for neural tissue engineering.

### 5.1 Bone and cartilage tissue

**Fig. 3:**
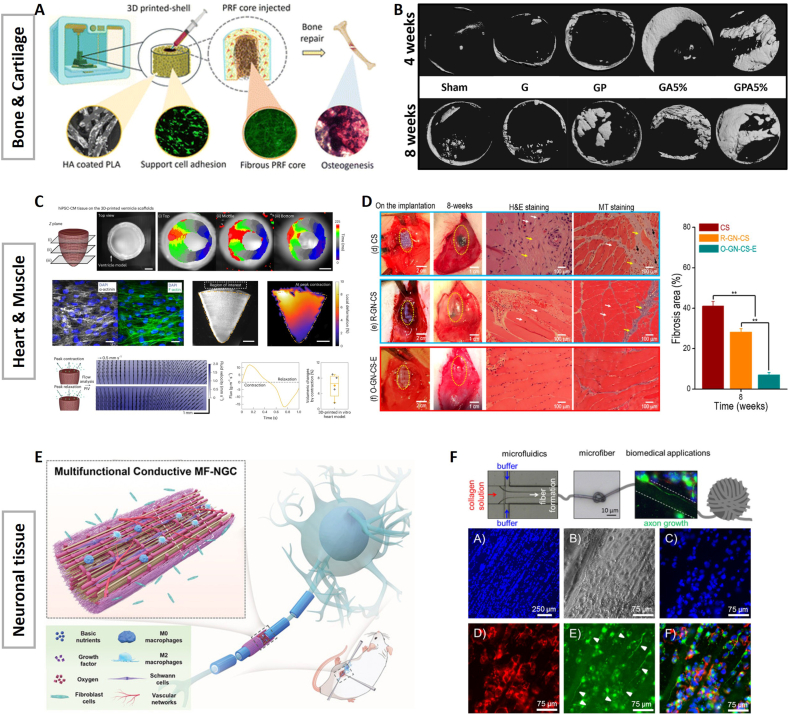
Applications of fiber integration. A Mimicry of the core-shell structure of bone by implementing two different hydrogels: a stiffer one for the shell and a softer one for the core. Adapted from Ref. [34]. B Bone recovery is positively impacted by fiber integration *in vivo*. Adapted from Ref. [30]. C Fiber integration increases muscle contraction in bioprinted, fiber-added ventricle. Adapted from Ref. [52]. D Kamaraj showed that fiber integration helps reducing fibrosis upon implantation. Adapted from Ref. [56]. E By implementing different size ranges of fibers (nano, micro) and hierarchically placing them, a nerve conduit was engineered to support peripheral nerve regeneration. Adapted from Ref. [32]. F Collagen microfibers were shown to support the neurite outgrowth *in vitro*. Adapted from Ref. [75].

Bone and cartilage tissue represent classic examples when it comes to load-bearing applications and fiber reinforcement is particularly advantageous. Consequently, most studies utilize synthetic fibers to enhance mechanical strength and biological performance. For instance, Kosik-Koziol et al. bioprinted an alginate-based bioink supplemented with electrospun PLA fiber fragments as a cartilage tissue precursor [10]. The fiber-filled constructs demonstrated structural stability over the course of 14 days and outperformed the fiber-free constructs in terms of cytocompatibility *in vitro*. Moreover, chondrogenic differentiation was supported, indicated by the expression of aggrecan and collagen type II in the fiber-filled constructs. In a separate study focused on personalized bone regeneration, Moghimi et al. developed a two-step bioprinting process mimicking the core-shell cancellous-cortical structure of bone [34] (Fig. 3A). Specifically, they employed hydroxyapatite (HA) coated PLA microfibers embedded in a GelMA-based matrix to create the stiffer shell while the soft core consisted of non-coated PLA microfibers and platelet-rich fibrin. This hierarchical construct promoted higher viability, cell spreading and migration and a significant increase in proliferation after 7 days. Notably, osteogenic differentiation markers as alkaline phosphatase and Alizarin Red staining (indicative of calcification) were significantly elevated, highlighting the role of different fiber size and surface modification in tailoring microenvironments to specific cell types. Dubey et al. further demonstrated the benefits of polycaprolactone (PCL) fiber reinforcement in combination with amorphous magnesium phosphate (AMP) for guided bone regeneration [30] (Fig. 3B). *In vitro*, the constructs exhibited improved mechanical stiffness, cytocompatibility and cell adhesion. The addition of PCL fibers led to higher levels of calcification and increased expression of osteogenic differentiation markers, including RUNX2 (core-binding factor alpha-1 Cbfa1), collagen type I and osteopontin. *In vivo*, using a bilateral critical size calvarial defect model in rats, PCL fiber supplementation resulted in an 8-fold increase in bone volume after 4 and 8 weeks, underscoring their regenerative potential. Overall, this emphasizes the beneficial use of fiber-reinforcement as an adjustable platform for bone and cartilage regeneration.

### 5.2 Cardiac and skeletal muscle tissue

A growing body of work has explored the anisotropic alignment of fibers to guide contractile tissue formation in cardiac and skeletal muscle tissue. In this regard, research focuses on natural and electrically conductive fiber materials. For instance, silk microfibers gained attention due to their robust structural support and shear-induced alignment possibility [56]. Here, bioprinting enabled the alignment in printing direction and an increased cell-matrix interaction demonstrated by cytoskeletal reorganization. Similarly, Choi et al. used short gelatin microfibers as biological cues for improved cardiac tissue engineering [52] (Fig. 3C). Extrusion bioprinting was used to build a 3D structure resembling the ventricular cavity and moreover achieve anisotropic alignment in printing direction. This resulted in the guided growth of cardiomyocytes along the gelatin fibers, thereby enabling biomimetic electrophysiological and contractile properties. Altogether, this study offers a promising strategy to overcome scale limitations—from micro-to centimeter scale—enabling intra- and extracellular organization like native heart tissue. Notably, Kim et al. translated previous *in vitro* results into *in vivo* models: collagen-based muscle patches incorporated magnetically aligned gold nanowires which enabled a high degree of myoblast alignment and efficient myotube formation [29] (Fig. 3D). Specifically, the engineered muscle tissue underwent complete transformation into mature myofibers. Eight weeks after implantation in rats, fibrosis and inflammatory cell infiltration were significantly reduced. These findings highlight the potential of fiber alignment strategies for restoring functional contractility in damaged tissue.

### 5.3 Neural tissue

Neural tissue regeneration is supported by a variety of biomaterials of which natural and synthetic polymers gained interest [103]. Electrospun fibers are widely used for assembling nerve conduits, offering control over fiber properties and hierarchical design. The integration of conductive polymers like polypyrrole (PPy) with silk fibroin (SF) has shown promise in creating electrically conductive scaffolds that support neural cell adhesion, proliferation, and differentiation [104]. These composite scaffolds demonstrate good biocompatibility and stability, with electrical conductivity ranging from 1 × 10^−5^ to 1 × 10^−3^ S/cm. When combined with electrical stimulation, PPy/SF scaffolds have been found to enhance axonal regeneration and remyelination *in vivo*, potentially through the activation of the MAPKs signal transduction pathway. In the same vein, Fang et al. developed a hierarchical conductive scaffold made by PCL electrospinning to model peripheral nerve injury [32] (Fig. 3E). The scaffold comprises three areas: the outer, nanofibrous layer is permeable for nutrient diffusion, the middle microfibrous layer promotes neuronal growth and neovascularization whereas the innermost conductive layer enables electroactive properties. By its advanced design, peripheral nerve injuries are regenerated *in vivo* suggested by improved axon myelination, muscle weight increase and sciatic nerve function index. Natural polymers as collagen provide better biocompatibility and have been widely used to guide neural cell growth. For instance, microfluidically spun collagen fibers with diameters of 8 μm promoted adhesion to the neuronal cell line NG108-15 [75] (Fig. 3F). Subsequent axonal outgrowth was guided along the fiber axis. Likewise, neuronal root ganglia as well as embryonic Schwann cells adhere to wet-spun collagen fibers and were able to build axons which aligned with the fiber orientation [50]. Additionally, cell migration and a dense morphology along the length of the fiber edge were seen. These results emphasize the potential of collagen fibers for peripheral nerve injury repair. Interestingly, when directly spinning neuronal precursor cells with collagen, incorporated cell bodies align in fiber direction due to the fibrillar anisotropy within the fiber [7]. By co-culture of endothelial cells neuronal differentiation markers as neurogenesis neurofilament-66 and beta3-Tubulin were increased hinting at the beneficial influence of a co-culture on nerve regeneration.

## 6 Summary and conclusion

Fiber integration offers a powerful strategy for adjusting the mechanical, structural, and biological properties of bioinks. Its influence depends heavily on fiber type, morphology, concentration and fiber-matrix compatibility. Synthetic fibers tend to have a mechanical reinforcing effect, while natural fibers have a higher degree of inherent biofunctionality. To be compatible with biofabrication techniques such as casting or bioprinting, fibers must be in a fragmented form with their size and length ranges tailored to nozzle geometry. Alternatively, biofabrication methods must be adapted to enable the processing of continuous fibers. The fragmentation of fibers allows for a higher degree of technological openness, but has weaknesses in terms of consistency, e.g., in their size and distribution. In contrast, continuous fibers require a defined application technology, but allow for new ways of functionalization, leading to the direct integration of long, continuous fibers with defined size and distribution. The application examples presented in chapter 5 illustrate the wide range of possibilities that fiber integration offers in terms of tissue engineering. Mechanically reinforcing fibers demonstrate their advantages in the cultivation of load-bearing tissues such as bone or cartilage. Moreover, fiber orientation, which is significantly influenced by biofabrication technology, plays a more important role in the production of (heart) muscle tissue. The implementation of additional functionalizations such as electrical conductivity, is essential for controlling nerve cell growth. By providing more advances *in vitro* models, fiber-reinforced hydrogels can help reduce reliance on animal experiments and enable more predictive studies of human biology in health and disease. They open the door to personalized regenerative therapies and engineered tissues tailored to patient-specific needs. These materials could support the development of 4D-metamaterials that adapt and remodel in response to their environment. For example, thermosensitive fibers could enable a time dependent folding of 3D structures. AI can be a powerful tool to optimize process monitoring, automatization as well as fiber-matrix compatibility design and the tailoring of fiber-cell interaction. The synergistic effects of AI and 3D printing were already discussed to produce large-scale, individualized organ models [105]. Recently, another study developed an open-source AI to model fiber-filled hydrogels to design fiber-reinforced hydrogels for specific applications [106].

However, when analyzing the extensive research data from the literature, a weakness inherent in research freedom became apparent. The inconsistent use of matrix and fiber materials, concentrations, and mixing ratios, as well as the different characterization methods and properties investigated, make in-depth quantitative analysis difficult. There are only single attempts to formulate design principles until now [86]. Thus, the effects of fiber integration can currently only be described in qualitative terms: 1) Fiber integration generally leads to an increase in shear viscosity (Fig. 2A). 2) Fiber integration can increase the mechanical properties (e.g., the modulus of elasticity) up to a matrix-dependent maximum value before a reduction occurs due to the increase in potential defect sites (Fig. 2B). 3) Fiber-matrix composites exhibit a non-linear stress-strain curve (Fig. 2C), which can be used to simulate the equally non-linear mechanical properties of native tissue such as blood vessels. 4) The fiber diameter influences the adhesion and polarization behavior of cells. Polarization initially increases with increasing diameter before subsequently decreasing (Fig. 2D). This effect is in turn dependent on cell size and must be considered relative to it.

It is currently not possible to make a generally valid statement about the quantitative influence of fiber integration on material behavior. This is primarily due to the widely scattered and non-standardized data landscape found in the analyzed publications (see Table 1, Table 2). The possible increase in the modulus of elasticity, for example, depends significantly on the stiffness of the underlying matrix material and its concentration. The influence of fiber integration based on a 0.5 % collagen matrix cannot be directly compared with that of a 2 % agarose matrix. Until now, there has been a lack of standardized data to establish general design guidelines. The latter would be necessary to be able to formulate theoretical models and recommendations for action as a design framework. Nevertheless, the trends described above, which can already be gleaned from the data, can be understood as a useful basis. With this basis, the general relationships can already be well transferred to given starting materials. Future research should focus on deeply understanding the interactions governing fiber-matrix compatibility to maximize mechanical and cellular output.

## CRediT authorship contribution statement

**Annabelle Neuhäusler:** Writing – review & editing, Writing – original draft, Visualization, Project administration, Funding acquisition, Conceptualization. **Nils Lindner:** Writing – review & editing, Visualization. **Andreas Blaeser:** Writing – review & editing, Supervision, Resources, Project administration, Funding acquisition, Conceptualization.

## Declaration of competing interest

The authors declare that they have no known competing financial interests or personal relationships that could have appeared to influence the work reported in this paper.

## Data Availability

No data was used for the research described in the article.
